# Delivery of Heterologous Proteins, Enzymes, and Antigens via the Bacterial Type III Secretion System

**DOI:** 10.3390/microorganisms8050777

**Published:** 2020-05-21

**Authors:** Heather A. Pendergrass, Aaron E. May

**Affiliations:** Department of Medicinal Chemistry, School of Pharmacy, Virginia Commonwealth University, Richmond, VA 23219, USA; pendergrassha@vcu.edu

**Keywords:** type III secretion system, heterologous protein secretion, vaccine delivery, secretion tags, fused proteins, pathogenesis, drug discovery, Gram-negative pathogens

## Abstract

The Type III Secretion System (T3SS) is a multimeric protein complex composed of over 20 different proteins, utilized by Gram-negative bacteria to infect eukaryotic host cells. The T3SS has been implicated as a virulence factor by which pathogens cause infection and has recently been characterized as a communication tool between bacteria and plant cells in the rhizosphere. The T3SS has been repurposed to be used as a tool for the delivery of non-native or heterologous proteins to eukaryotic cells or the extracellular space for a variety of purposes, including drug discovery and drug delivery. This review covers the methodology of heterologous protein secretion as well as multiple cases of utilizing the T3SS to deliver heterologous proteins or artificial materials. The research covered in this review will serve to outline the scope and limitations of utilizing the T3SS as a tool for protein delivery.

## 1. Introduction

**Figure 1:**
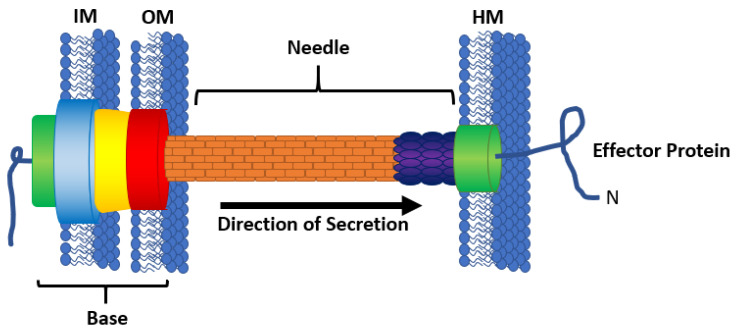
The bacterial type III secretion system (T3SS). The base of the T3SS anchors the apparatus into the bacterial inner membrane (IM) and outer membrane (OM), and is composed of an ATPase (green), the inner ring (blue), the outer ring (red), and a connecting channel (yellow). The needle (orange) spans the intercellular space between the bacterial cell membranes and the eukaryotic host membrane (HM). The translocon (green) forms a pore in the HM. Effector proteins (blue line) travel through the needle from the bacterial cell into a host cell, leading with the N-terminus.

The bacterial Type III Secretion System (T3SS) injectisome is a multimeric protein complex composed of more than 20 different proteins to form a needle-like structure to transverse the space between an interacting bacterial cell and eukaryotic cell (Figure 1) [1,2,3]. The base of the T3SS is composed of inner and outer rings that anchor the needle into the bacterial membranes and an ATPase responsible for powering secretion [4,5,6]. Some of the secreted proteins oligomerize to form a needle with an inner diameter of approximately 2.5 nm [3,7,8]. Because of the small size of the needle, all proteins that transverse the T3SS must be linearized or unfolded before passage. The tip of the needle, or the translocon, forms a pore in the eukaryotic cell membrane through which effector proteins may travel [3,9]. Once inside the host cell, host folding enzymes may be recruited to aid in refolding of the linearized effectors. These effector proteins elicit effects including but not limited to intimate attachment of the bacteria to the host cells [10,11], reprogramming of host cell machinery [12,13], evasion of immune response [14], or cell death [15]. For pathogenic bacteria, an active T3SS aids in propagation of infection, and an inactive T3SS results in attenuation of infection [16,17].

Since its discovery, the T3SS has been implicated as a virulence factor utilized by certain Gram-negative pathogens such as enterohemorrhagic and enteropathogenic *Escherichia coli* (EHEC and EPEC, respectively) [10,18,19], *Salmonella enterica* serovar Typhimurium [20,21], *Chlamydia* spp. [22,23], and *Yersinia pestis* [24,25,26], the causative agent of the plague. Each year, these pathogens infect more than 2 million people in the United States [27,28,29,30]. Pathogens with nonfunctioning T3SSs are often rendered avirulent [16,31,32]. In addition, murine models indicate that inhibition of the T3SS by small-molecule inhibitors results in attenuation of infection [17,33]. Bacterial cells are still viable when the T3SS is inhibited or rendered nonfunctional [34]. As a result, resistance to T3SS inhibitors should develop more slowly than resistance to traditional antibiotics [35]. Characterization of the T3SS is a continuing focus of study for researchers interested in understanding mechanisms of bacterial pathogenesis and the inhibition of this complex as an anti-infective strategy.

In recent years the secretory function of T3SSs has been explored for delivery of antigens in vaccination and as a tool for the production and secretion of heterologous proteins [36,37,38]. To better understand the applicational capabilities of the T3SS, the expression and secretion of heterologous proteins has been investigated. For example, the T3SS-utilizing phytopathogenic strain *Pseudomonas syringae* pv. *tomato* DC3000 has had the genes for its effector proteins removed [39,40,41,42]. This resulting strain (DC3000D36E) was avirulent but maintained a functional T3SS injectisome. Other T3SS-encoding bacteria with attenuated virulence have been researched for their potential as drug delivery machines [37]. When perfected, this technology may be utilized to combat the difficulties associated with using proteins as drugs or probes in humans. Since proteins are secreted directly into human cells via the T3SS, the characteristic issue of proteins permeating cell membranes would be circumvented, and proteins could be delivered directly to target cells. In this review, we will give an overview of the methodology for heterologous or non-native protein secretion via the T3SS, and cover examples of the application of these technologies.

## 2. Strategies for the Secretion of Heterologous Proteins

**Figure 2:**
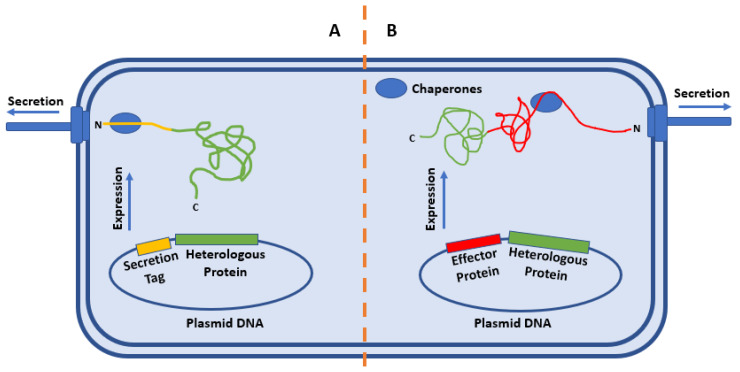
Strategies of heterologous secretion. (**A**) Conjugation of the conserved secretion-enabling sequence for the organism to the protein of interest will result in recognition of the protein for secretion and translocation through the T3SS machinery. (**B**) Conjugation of the protein of interest with a native effector protein will result in targeting of the protein of interest to the T3SS machinery in an unfolded state and allow for translocation via the T3SS.

Two methods are typical for targeting heterologous proteins for secretion: labeling the protein with a secretion tag (Figure 2A) [43] and fusing a heterologous protein to a native effector (Figure 2B) [44]. Regardless of which method is being used, the nature of the relationship between the tag or effector protein with its native chaperone must be considered. In some cases, secretion of effectors is reliant on the presence of the concordant chaperone. In other cases, the presence of the chaperone can increase cellular levels of the effector proteins or increase the basal level secretion of a heterologous protein. If the secretion of the effector is dependent on a chaperone, the chaperone binding domain must be present in the tagged or fused protein for successful secretion.

### 2.1. “Secretion Tag”

Some effectors that are secreted via the T3SS encode an N-terminal tag that is recognized by an autoprotease located at the base of the needle [43,45,46]. In the bacterial cytosol, designated chaperone proteins bind to the N-terminus of the effectors [5]. This is thought to aid in the stability of the effectors, improving cytosolic accumulation [47], and chaperone binding prevents effectors from folding, keeping the N-terminus linearized for transport to the base of the T3SS [48]. Once the tag is recognized, the ATPase located at the base of the injectisome powers the rapid translocation of the unfolded protein through the needle [6]. The rate of protein secretion has been measured at 7 to 60 proteins per cell per second in *Salmonella* [49]. By adding the same secretion tag to the N-terminus of a heterologous protein, it can be recognized by the autoprotease within the sorting platform and secreted via the T3SS. In some pathogens, such as *E. coli*, the secretion tag on each effector is different, and some tags are secreted more efficiently than others, making some secretion tags a better choice for heterologous protein secretion. It is speculated that the different efficiencies between effector tags allow for hierarchal secretion of effectors in a specific order [43].

**Figure 3:**
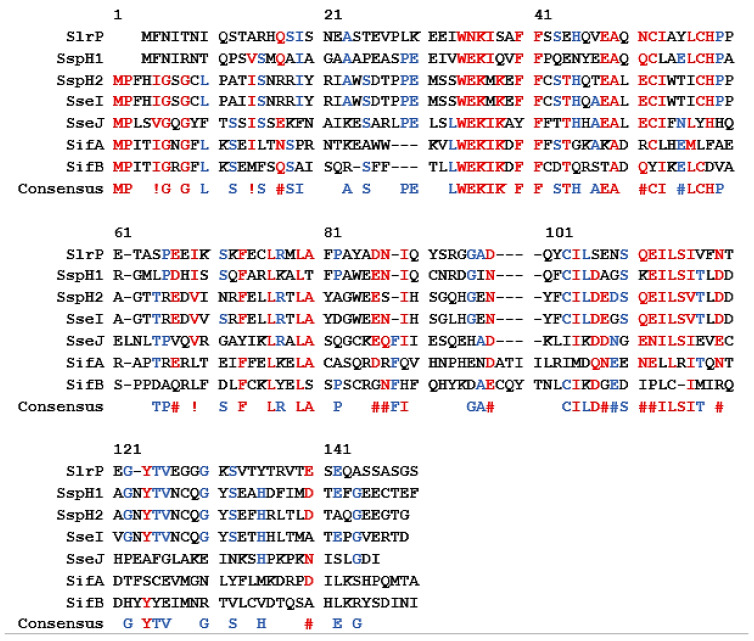
Figure adapted from [50]. Alignment of seven effector and translocator proteins involved with the T3SS of *Salmonella typhimurium*. Red residues are high consensus; at least 5 of 7 proteins contain these residues. Blue residues are low consensus; 3 or 4 of seven proteins contain these residues. Black residues have no consensus. Similar residues are marked as following: #, N D Q or E; !, I or V.

A consensus sequence for the identification and secretion of effector proteins in *Salmonella* Typhimurium was published by Miller et al. in 2000 (Figure 3) [50]. Based on alignment studies, the amino-terminal 150 amino acid residues show sequence similarity between 7 selected effector proteins. In addition, other regions of the amino acid sequence also indicate homology between the effectors. The minimal translocation sequence for one protein, SspH2, was determined using a translocation assay. The *Bordetella pertussis cyaA* gene was fused to the amino-terminal consensus sequence of SspH2 (SspH2-CyaA), resulting in the expression and secretion of a tagged CyaA adenylate cyclase toxin. After translocation into a eukaryotic cell, CyaA converts ATP to cAMP and increases in cAMP concentration can be measured. Results indicated that translocation efficiency decreases if fewer than 214 amino acids are present within the secretion tag, but secretion is still possible. If the highly conserved amino acids 32 to 35 are absent, then no translocation of the fusion protein was observed. This indicates the importance of using the correct sequences in characterization or screening assays. For each organism being observed, the necessary secretion tag must be identified. 

The differences in secretion tags between effectors are important for translocation efficiency. Munera et al. conducted a study in which secretion tags from *E. coli* were analyzed for their efficiency in the secretion of β-lactamase TEM1 [43]. The first 20 N-terminal amino acids from several effector and translocator proteins as well as a random 20 amino acid sequence were conjugated to the N-terminus of TEM1. Secretion was observed by SDS-PAGE and analyzed with an assay that quantified TEM1 activity post translocation into mammalian cells. The random control allowed no secretion of TEM1, but each tag from the effector proteins enabled the secretion of TEM1 into mammalian cells. The group reported the efficiency of each tag, with EspF being the most efficient.

### 2.2. Whole-Protein Fusions

Recognition is not the only challenge to be overcome for successful heterologous protein secretion. As mentioned above, proteins to be secreted must be unfolded to be able to pass through the needle of the T3SS [3,7,8]. The presence of the secretion tag notwithstanding, if the N-terminus of the protein folds before it is passed through the secretion system, it may not be translocated. Conjugating the protein of interest to an existing effector directly combats this problem (Figure 2B). While the chaperone binding domain of some effectors is located near the N-terminus of the effector protein, the chaperone binding domain can occur anywhere within the structure of the effector [47]. The location of the chaperone binding domain is important with respect to premature folding, effector stability, and translocation. When a heterologous protein is expressed in conjugation with an effector, the effector, secretion tag, and associated chaperones can increase translocation efficiency.

The importance of effector/chaperone pairing was evidenced by work from Zurawski et al. [51]. The *S. enterica* protein SseA is a chaperone for the translocon protein SseB [51]. To study the chaperone/effector relationship, SseB was fused to the N-terminus of the heterologous enzyme CyaA (SseB-CyaA). CyaA increases cAMP levels in eukaryotic cells when secreted via the T3SS, which can be readily quantified. When the chaperone SseA was present, SseB-CyaA successfully passed through the T3SS. In strains lacking the SseA chaperone, SseB was not secreted and cAMP levels were unchanged in the host cell. These results highlight the importance of chaperones and validates the use of existing effectors for secretion of heterologous proteins.

## 3. Physics of Substrate Secretion

### 3.1. Secretion Efficiency

In 2009 Widmaier et al. expressed and secreted spider silk monomers from *S. typhimurium* for the purpose of characterizing the physics of secretion [52]. *S. typhimurium* encodes two T3SSs, each in separate regions of the genome. These gene groups are referred to as *Salmonella* Pathogenicity Islands 1 and 2 (SPI-1 and -2) [21,53]. In this study, the genes for three kinds of spider silk monomers ADF-1, -2, and -3, having molecular weights of ~30 kDa, ~25 kDa, and ~56 kDa, respectively, were fused to the N-terminal secretion tag of effector SptP and expressed under the same operon with chaperone protein SicP [52]. This emulates the native configuration of these proteins within SPI-1. Previous research suggests that successful accumulation and secretion of SptP is dependent on the presence of SicP [48]. The silk monomers were also tagged on the C-terminus with FLAG for Western blot visualization. 

The efficiency of secretion of ADFs 1-3 into the supernatant was determined to be 9%, 17%, and 7.6%, respectively, of the total expressed, meaning that the majority of the ADF remained within the cell, unsecreted (Table 1) [52]. Despite the inefficient secretion, secretion rates of up to 1.8 mg L^−1^ hr^−1^ were measured using flow cytometry. The highest concentration of secreted protein was detected 6 h after initiation of secretion. The authors found no evidence of “leaky secretion,” meaning that secretion of these proteins was initiated by activation of the T3SS. In the absence of either the chaperone or the secretion tag, the effectors were not visible by Western blot. These results highlight the critical importance of using a secretion tag on a heterologous protein and that challenges still remain in improving the secretion efficiency.

The T3SS works rapidly to secrete effector proteins [49,55,56]. For example, *Yersinia pseudotuberculosis* will secrete effector phosphatase YopH within 30 s of the onset of infection (Table 1) [55]. YopH is responsible for dephosphorylation of phosphotyrosine proteins that enhance phagocytosis. This reaction is an important aspect of T3SS-mediated infection that allows *Y. pseudotuberculosis* to evade host response. Studies on *Shigella flexneri* indicate that cytosolic stocks of effectors IpaB and IpaC are secreted soon after initiation of T3SS signaling, with 50% of the protein secreted within 240 s (Table 1) [56].

Metcalf et al. developed a strategy for synthetically upregulating T3SS activity [54]. The native transcriptional regulator HilA activates the *S. typhimurium* SPI-1 T3SS. Overexpression of HilA was achieved by using a plasmid, whereby HilA was overexpressed in the presence of isopropyl-β-D-thiogalactopyranoside (IPTG). The high cytoplasmic concentration of HilA resulted in increased expression of the T3SS as indicated by Western blotting. The secreted protein titer was measured via quantitative Western blotting. A titer of over 28 mg/L was measured for one unspecified protein of human origin (DH, Table 1). This was a 10-fold increase compared to titers observed for samples without overexpressed HilA. Metcalf et al. also reported that changing concentrations of IPTG resulted in concordant changes in T3SS activity, indicating sensitivity and opportunity for optimization of this protein secretion strategy.

The secretion rate of T3SS1 in *S. typhimurium* was quantified by Schlumberger et al. [49]. The T3SS1 responds rapidly to initiation signals, secreting effector SipA into target cells within 10–90 s. The secretion of SipA continued for 100–600 s until cytosolic concentrations of the effector were depleted (Table 1) [49]. The rate of secretion was estimated at 7–60 molecules of SipA per second per cell (5000–50,000 amino acids per second per cell). It was unclear how many secretion apparatuses were present on the cell surface and how many of them were participating in secretion [49]. For comparison, the typical translational elongation rate in bacteria ranges from 4–22 amino acids per second [57,58,59]. While the T3SS in *Salmonella* rapidly secretes effectors within minutes of initiation of secretion, Widmaier et al. showed evidence that secretion by this organism may continue for up to 6 h [52]. This allows abundant time for protein accumulation in the supernatant.

### 3.2. Protein Folding

In 2014 Dohlich et al. used a knotted fusion protein to validate the theory that secreted proteins are unfolded for translocation by *S. flexneri* [60]. IpaB is an effector protein of *Shigella* that causes pyroptosis in macrophages. The C-terminus of IpaB was fused to a knotted protein, exposing the N-terminal region of IpaB (IpaB-knot). This was done to confirm the theory that the N-terminal domain of effectors is secreted first. The function of the IpaB-knot fusion was confirmed in a macrophage lysis assay, which showed that the fused protein maintained pyroptotic activity. The IpaB-knot fusion was co-purified with needle complex proteins after centrifugation, as confirmed by Western blot using anti-IpaB and anti-MxiG antibodies to visualize the fusion protein and the needle complex proteins, respectively. Immuno-electron microscopy was then used to visualize the IpaB-fusion protein within the needle complex. The fusion was constructed with a flanking Strep tag on the N- or C-terminus of the fusion protein (Strep-IpaB-knot or IpaB-knot-Strep). Using anti-Strep antibodies, Dohlich et al. visualized the Strep tag on both the tip of the needle and at the base for the amino tagged or carboxyl tagged constructs, respectively. This confirmed that the N-terminus of the IpaB peptide passed through the needle unfolded and could not pass further due to the knotted peptide on the C-terminus. This result also indicates that the T3SS is not capable of flowing in reverse or bringing effectors back within the bacterial membranes if passage through the needle is obstructed.

T3SS chaperones have multiple functions, including increasing the stability of an effector protein after it is expressed [47]. This allows for accumulation of the effector within bacterial cytosol until secretion. Niebuhr et al. showed that the *S. flexneri* effector IpgD requires the presence of its chaperone IpgE for cytosolic accumulation [61]. When a nonpolar mutant of chaperone IpgE was generated to eliminate coordination with IpgD, IpgD concentrations in supernatant samples and in cell lysate decreased, and no bands for IpgD were visible by Western blot. The band was restored when the wild type IpgE chaperone was expressed on a plasmid. It was hypothesized that chaperones maintain an extended conformation of the N-terminus of effectors before they are translocated [47,48,62].

Structural analysis of the interactions between *S. typhimurium* SptP and SicP indicate that the chaperone binding domain of SicP is held in an extended conformation by chaperone SptP [48]. Meanwhile, the C-terminus folds into its final globular conformation, which is unfolded before secretion. When the heterologous protein Lis was conjugated to effector SopE in *S. typhimurium* (SopE-Lis), its secretion was increased when it was also conjugated to chaperone protein InvB (InvB-SopE-Lis) [63]. Widmaier et al. optimized the secretion of the spider silk monomers ADF 1–3 in *S. typhimurium* by altering combinations of effectors and chaperones in order to determine the most efficient paring [52]. In some cases, effector secretion is not reliant on a chaperone, but, when attempting the secretion of a heterologous protein, optimization of the effector/chaperone combination and ratio may improve secretion efficiency.

Once proteins pass through the T3S injectisome, they must refold into the correct conformations. This process has been analyzed in the interest of protein production and isolation [38]. During a typical protein production process, proteins accumulate within the cytosol of bacterial cells or within inclusion bodies within the cytosol. The proteins of interest are then purified from the amassed proteins within the bacterial cells after lysis. Secretion through the T3SS could serve as a useful strategy for protein purification, as the protein of interest can be selectively transported to the supernatant of the bacterial culture, removing it from the bacterial proteome; however, since secreted proteins are necessarily unfolded before T3SS-mediated transport, correct folding of the protein is required in the supernatant. The extent of protein refolding in the extracellular space was quantified by Metcalf et al., who compared the refolding of two different enzymes, β-lactamase EC:3.5.2.6 and the alkaline phosphatase EC 3.1.3.1, after secretion [38].

The β-lactamase, EC:3.5.2.6, class A, is a monomeric enzyme with one disulfide bond that is not required for enzymatic function [38]. In addition, no cofactors are required for enzymatic activity. Catalytic activity was observed after secretion into standard LB media, and the fraction of correctly folded protein was assumed to correlate with activity. By comparing the total amount of enzyme in the supernatant to the activity of the supernatant samples, they estimated that ~15% of the secreted enzyme was catalytically active. When the NaCl concentration in the supernatant was increased from 5 g/L to 17 g/L, the fraction of catalytically active protein increased 3-fold. This indicated that culture conditions may be optimized for protein refolding based on the required environment of the protein of interest.

Alkaline phosphatase (AP) EC 3.1.3.1, isozyme 1, is a dimeric enzyme that requires the acquisition of one Mg^2+^ ion and two Zn^2+^ ions, as well as the formation of two disulfide bonds for enzymatic activity [38]. Enzymatic activity was observed in secreted samples, indicating that disulfide bond formation had occurred to produce the catalytically active conformation. Individual mutation of each of the four cysteine residues that participate in disulfide bond formation resulted in inactive enzymes after secretion. Measurement of enzymatic activity indicated that ~85% of the secreted enzyme was active and correctly folded. This shows that it is possible for secreted enzymes to dimerize and acquire the necessary cofactors for correct folding after secretion, even in the absence of cellular folding enzymes. Increasing the salt concentration to 17 g/L, however, decreased the amount of correctly folded and catalytically active enzyme to ~60% of the total secreted. These data validate the use of the T3SS as a protein purification strategy and indicate the customizable nature of this method.

The results from these studies with artificial substrates enhances our understanding of the scope of which heterologous proteins can be secreted into and become catalytically active within the supernatant. The rate of protein secretion was confirmed, and an expression/secretion ratio was established by Widmaier et al. [52]. Widmaier et al. also characterized the relationship between chaperones and secretion tags in *Salmonella*. Dohlich et al. visualized a T3SS apparatus in the process of secreting a protein and confirmed the hypothesis that the N-terminus of the T3SS is secreted first [60]. Metcalf et al. quantified the fraction of correctly folded enzymes with varying cofactor and disulfide bond requirements for enzymatic activity after secretion and showed dimerization is possible after secretion [38]. The research has also shown that eukaryotic protein such as silk monomers can be secreted via the T3SS, and it is possible to block the T3SS, rendering it inactive. These results have important implications for what is possible using the T3SS in protein production and purification.

## 4. Delivery of Antigenic Proteins

It has been recognized that the T3SS could be used to deliver antigenic peptides for the purpose of vaccination [64]. This strategy uses heterologous protein secretion via the T3SS to translocate antigens to intracellular compartments necessary to stimulate an immune response. One pathogen popular for this purpose is attenuated *S. typhimurium* [37,63,64,65,66]. This organism has been used as a delivery system for many antigenic proteins from pathogenic bacteria and viruses alike. In each case, the antigenic proteins or epitopes were fused to a secretion tag or a full effector encoded by one of the two SPIs to prime it for secretion.

A representative case for antigen secretion involves the secretion of SaEsxA and SaExcB via the T3SS to vaccinate against *Staphylococcus aureus* infection [65]. The genes for SaEsxA and B were fused to the secretion tag of effector SipA in the SPI-1 of *S. typhimurium* (SipA-SaEsxA and -B). Translocation of the His-tagged antigens into the cytosol of macrophages was confirmed via Western blot with anti-His antibodies. In vivo studies were conducted with BALB/c mice to determine the resulting immune response. Antigen-specific immune responses were analyzed with ELISA and ELISPOT assays 7–9 days after immunization. The mice showed increased survival in response to a lethal challenge by *S. aureus* after immunization compared to a vector control strain. A similar study used the fused protein technique, fusing the antigen PcrV of *Pseudomonas aeruginosa* to the full-length protein SseJ of *S. typhimurium* (SseJ-PcrV) [66]. In this case, mice were immunized 21 days prior to challenge by *P. aeruginosa*. 60% of the immunized mice survived the challenge while none of the nonimmunized mice survived.

Rüssmann et al. successfully delivered influenza nucleoprotein (IVNP) viral epitopes to murine RMA thymoma cells via the *S. typhimurium* T3SS [64]. The gene for the influenza NP epitope was hybridized with SptP to create chimeric protein SptP-IVNP. The translocation of the epitope to the cytosol of murine RMA cells resulted in presentation of the viral epitope to class I-restricted T cell hybridoma, as confirmed by measurement of the interleukin-2 (IL-2) secretory response from the T cells. In the following in vivo experiments, C57BL/6J mice were orally inoculated with avirulent *S. typhimurium* expressing SptP-IVNP. After inoculation, the mice began to produce cytotoxic T lymphocyte (CTL) precursors. These precursors were capable of lysing target cells infected with influenza or loaded with synthetic NP. In a following study, BALB/c mice were orally dosed avirulent *S. typhimurium* expressing a SptP hybrid with an NP from murine lymphocytic choriomeningitis virus (SptP-LCMVNP). These mice were protected from a lethal dose of LCMV.

While the concept of utilizing T3SS-harboring avirulent bacteria to deliver epitopes for vaccination has been established, the method of using live and replicating bacteria for vaccination may be incompatible for immunocompromised patients or children. For this reason, Carleton et al. developed a vaccination method using nonreplicating minicells with T3SSs [63]. Minicells are the result of unsuccessful cell replication, and do not contain chromosomal DNA, but they maintain the ability to produce protein and maintain the proton gradient necessary for ATP-dependent energy expenditure. They are also still capable of stimulating an innate immune response, which is important in this context. *S*. Typhimurium cells with a deletion in the *minD* gene are prone to aberrant replication and produce large amounts of minicells. These cells were found to produce the T3SS as encoded by the first *Salmonella* Pathogenicity Island (SPI-1) at concentrations lower than that of replicating bacterial cells. The group transformed the bacteria with a plasmid encoding HilA, a positive transcriptional regulator of SPI-1. This resulted in the necessary upregulation of the T3SS. After the ability of the cells to produce and translocate effector proteins into eukaryotic cells was confirmed, an effector-antigen chimera was developed. The antigen OVA, which contains a class I-restricted peptide and a C-terminal FLAG tag, was conjugated to the secretion signal from SopE (SopE-OVA). A plasmid encoding this chimera was transformed into the minicells, and the translocation of the protein product into murine RMA cells was monitored. Successful translocation resulted in an MHC class I-restricted immune response. In vivo studies showed these minicells are capable of activating CD8^+^ T-cells. Secretion was then optimized with the addition of chaperone InvB to the plasmid producing SopE-OVA.

The ability of minicells to elicit a protective immune response was analyzed by stimulation of ex vivo bone marrow-derived dendritic cells [63]. The minicells in this study were transformed with a plasmid harboring the gene for a chimeric protein InvB-SopE-Lis, a combination of the chaperone InvB, the secretion tag of SopE and immunogenic peptides from listeriolysin O and p60 from *Listeria monocytogenes*. The stimulated dendritic cells were transferred by tail vein injection to mice. After 6 days, the mice were challenged with *L. monocytogenes*, and 3 days after challenge, mice were killed and the colony forming units (c.f.u.) of *L. monocytogenes* in the spleen was counted. Dendritic cells stimulated by minicells harboring the InvB-SopE-Lis plasmid with an active T3SS induced a protective immune response capable of lowering the c.f.u. of pathogenic bacteria within the spleens of the mice compared to control.

Overall, these results show the potential power of using T3SS-encoding bacteria to deliver antigens as a vaccination method. By utilizing heterologous protein secretion techniques, researchers have verified that bacteria with attenuated pathogenicity may be used to induce a protective immune response in animals. The potential toxicity associated with dosing replicating bacterial cells may be circumvented by the surrogate use of minicells that harbor the T3SS.

## 5. Translocation vs. Secretion

The secretion of proteins by the T3SS can be monitored by observing either translocation into a host cell or secretion into the supernatant [13,31,65,67]. The important difference between translocation and secretion lies in the presence of folding enzymes. When a peptide is translocated into a host cell, host folding enzymes can be recruited to help fold the protein into the correct conformation, while these enzymes are absent in the extracellular space [68]. These folding enzymes have little effect on outcome when detection methods are used that do not rely on correctly folded protein (for example, ELISA or Western blotting), but if detection is dependent on folding, translocation may be required.

Many high throughput screening campaigns for inhibitors of the T3SS rely on secretion rather than translocation for simplicity of experimental design. Yount et al. secreted and accumulated carboxypeptidase G2 (CPG2) in the extracellular space before analysis [67]. CPG2 is a metalloprotease dependent on moderate concentrations of zinc for proper folding and enzymatic activity [69]. The group reports diluting supernatant samples 1:9 with a buffer specifically designed to aid in CPG2 folding before attaining any readings in order to achieve a better signal [67]. This dilution results in lower concentrations of enzyme in the samples and ultimately dilutes the maximum signal compared to an undiluted sample.

Some high throughput screening methods rely on T3SS-mediated translocation of effectors. A hemolysis assay for analyzing the T3SS activity of enteropathogenic *E. coli* (EPEC), for example, involves the translocation of effectors EspB and EspD into the plasma membrane of erythrocytes [9]. These two pore-forming proteins open holes in the plasma membrane of the erythrocytes, and subsequent release of hemoglobin may be measured by absorbance. The T3SS inhibitory activity of the natural product aurodox, a polyketide produced by *Streptomyces* sp. K06-0806, was characterized using this method and was later confirmed in vivo [16].

## 6. Enzyme and Analyte Secretion for Monitoring T3SS Activity

Enzyme or analyte secretion are common methods for monitoring T3SS activity. These methods can be used for the identification of inhibitors of the T3SS [31,70,71,72,73]. In both cases of translocation into a host cell and secretion into extracellular space, the correct folding of the enzyme being secreted is important and must be considered. As described above, in experiments involving the expression of spider silk monomers from a plasmid, only about 14% of the expressed protein is secreted. When secreting enzymes, the activity of the enzyme after secretion varies due to differing amounts of spontaneous and correct folding [38]. This section will review a few established methods for analyzing T3SS activity using enzyme or analyte secretion. Many reviews exist that provide more information on specific inhibitors analyzed in vitro [25,72,73].

### 6.1. CyaA Translocation

Translocation of the heterologous enzyme CyaA via the T3SS results in cAMP formation in eukaryotic cells. CyaA is a calmodulin-dependent enzyme and is only activated once translocated into host cytoplasm that contains calmodulin. This method, referred to as the adenyl cyclase (AC) reporter system, was developed in 1994 by Cornelis et al. [13], who measured the increases in cAMP levels to demonstrate protein secretion by *Y. pestis*. More recently, the AC reporter system has been adapted for work in *E. coli* to confirm the translocation of effector EspB [74]. CyaA was fused onto the C-terminus of EspB (EspB-CyaA), and translocation of the effector was monitored. Crawford and Kaper showed that translocation of CyaA into HeLa cells was possible using the secretion tag for the *E. coli* effector Tir (residues 1–15). Since these initial studies, multiple effectors have been identified across different organisms after fusion of CyaA to hypothesized proteins.

Cardenal-Muñoz et al. created a library of fused effector SteA proteins for analysis of secretion and translocation of this effector under conditions that induce both the SPI-1 and SPI-2 encoded T3SSs in *Salmonella* [53]. A FLAG tag was fused to the C-terminus of SteA (SteA-FLAG) and secretion was analyzed via Western Blot using anti-FLAG antibodies for visualization. Secretion of SteA was observed under conditions that would induce T3SS1 and T3SS2 in *Salmonella*. SteA was fused to the catalytic domain of CyaA and translocation into HeLa cells was observed. Translocation was rapid, with cAMP concentrations peaking within 15 min. This conclusion is validated by the decreased invasion with a *prgH* mutant strain. PrgH is an essential protein for T3SS1. Concordantly, translocation was decreased in a *ssaV* mutant strain (ssaV is essential for T3SS2), indicating that T3SS2 plays a role in invasion of HeLa cells as well.

### 6.2. β-Lactamase (Bla) Translocation

Translocation of fused β-lactamase (Bla) proteins has been performed to analyze T3SS activity [44,75,76]. Once translocation of the fused Bla proteins has occurred, host cells are typically stained with a prefluorogenic substrate of the enzyme, which is then acted upon by the Bla, producing a fluorescent signal that may be quantified. Schesser et al. fused Bla to the C-terminus of native effector proteins YlrA, YlrB, and YlrC of *Y. pestis* and confirmed their translocation into host cells during infection [77]. Similar studies in *Yersinia* have also been performed to confirm that the pathogen is capable of infecting immune cells, which has implications for mechanism of infection [78].

Pan et al. performed a high throughput screen to discover new small molecule inhibitors of the *Yersinia* T3SS [25], and visualized results from their hits using a Bla translocation assay. The gene for Bla was fused to effector YopE (YopE-Bla). This strain was incubated with HeLa cells in the presence and absence of candidate small molecules. Translocation of YopE-Bla to HeLa cells was visualized after the addition of substrate CCF2-AM. The resulting images of the infected and uninfected HeLa cells were visualized by fluorescence microscopy. The concentration-dependent inhibition of T3SS activity by four lead compounds from their screen was observed, and morphological changes as a result of T3S are evidenced in the images.

### 6.3. Phospholipase Secretion

Phospholipases are attractive enzymes for HTS for T3SS inhibitors, as the addition of a cleavable substrate causes increases in fluorescent signal if the enzyme has been secreted [79]. This system does not require translocation into a host cell because the addition of the cleavable substrate is not dependent on host cell machinery. This brings into question the efficiency of folding of enzymes in extracellular space, as host folding enzymes are not present to increase folding efficiency.

Felise et al. developed an HTS assay using a fusion of the catalytic domain of phospholipase A2 and the SipA (SipA-PL2) effector from *S. typhimurium* [79]. The phospholipase activity of the secreted fusion protein acted upon the substrate N-((6-(2,4-dinitro-phenyl)amino)-hexanoyl)-2-(4,4-difluro-5,7-dimethyl-4-bora-3a,4a-diaza-s-indacene-3-pentanoyl)-1-hexadecanoyl-sn-glycero-3-phosphoethanolamine, or PED6. When PED6 was added to culture supernatant and cleaved, an increase in absorbance occurred, indicating an active T3SS. When absorbance measurements remained unchanged, the T3SS was inactive.

## 7. Conclusions

Delivery of heterologous proteins via the T3SS is a promising technique, as evidenced by the delivery of vaccines, discovery of new anti-virulence drugs, and biochemical analysis of the T3SS. Issues related to proper folding and efficient delivery of protein via the T3SS will need to be overcome to broaden the application of this method. This includes the ~14% secretion of expressed protein [52] and varying degrees of enzymatic activity after secretion [38]. This technique has been beneficial in identifying T3SS inhibitors. For medical research, the T3SS poses as an important target for mitigating virulence in pathogenic bacteria that infect over 2 million of patients every year [27,28,29,30], and nonpathogenic strains may be designed to use the T3SS as a delivery mechanism for vaccination or protein therapy [64]. The T3SS may also aid in protein purification processes by separating a protein of interest from the intracellular proteome [38]. The effort required to perfect this technology notwithstanding, the T3SS stands to serve varying facets of the research community.

## Figures and Tables

**Table 1:** Selected examples of secretion efficiency.

Organism	Protein	Heterologous (Y/N)	Data	Rate	Ref
*S. typhimurium*	ADF-1	Y	9% protein secreted	1.8 mg L^−1^ hr^−1^ Max conc. at 6 hr.	[52]
	ADF-2	Y	17% protein secreted		
	ADF-3	Y	7.6% protein secreted		
	SipA	N	Initiation within 10–90 s.	7–60 molecules sec^−1^	[49]
			Active secretion for 100–600 s.		
	DH	Y	28 mg L^−1^ titer		[54]
*Y. pseudotuberculosis*	YopH	N	Initiation within 30 s		[55]
*S. flexneri*	IpaB	N	~50% secreted within 240 s		[56]
	IpaC	N			
